# Analysis of the Permeability Capacity and Engineering Performance of Porous Asphalt Concrete

**DOI:** 10.3390/ma18174200

**Published:** 2025-09-08

**Authors:** Huan Wang, Lintao Li, Zebang Deng, Pengguang Liu, Dingbang Wei

**Affiliations:** 1 Guangdong Road and Bridge Construction Development Co., Ltd., Guangzhou 510635, China; 2 Guangdong Hualu Transport Technology Co., Ltd., Guangzhou 510550, China; 3 Gansu Industry Technology Center of Transportation Construction Materials Research and Application, Lanzhou Jiaotong University, Lanzhou 730070, China; 4 Gansu Transportation Planning, Survey and Design Institute Co., Ltd., Lanzhou 730030, China

**Keywords:** porous asphalt concrete, porosity, effective porosity, water permeability coefficient, engineering performance

## Abstract

This study investigates the permeability performance and engineering performance of porous asphalt concrete (PAC) mixtures. PAC-10 and PAC-13 mixture specimens with various porosities were prepared. The relationships among porosity, effective porosity, and effective porosity proportion were analyzed, and the pavement engineering performance was evaluated. Moreover, the effects of nominal maximum aggregate size (NMAS) and porosity characteristics on the permeability coefficient were also examined. The results indicate that both the effective porosity and the effective porosity proportion increase with total porosity for both the PAC-10 and PAC-13 mixtures. PAC-13 consistently exhibits a higher effective porosity than PAC-10, suggesting enhanced drainage performance. The designed PAC mixtures satisfy the requirements of high-temperature stability and moisture resistance for asphalt pavements, while the large porosity is contradictory with high-temperature stability and moisture resistance. Additionally, the permeability coefficient significantly increases with larger NMAS, and a strong linear correlation is observed between permeability and both total and effective porosity, where the coefficient of determination (R^2^) is larger than 0.9. These findings demonstrate that porosity parameters can serve as reliable indicators for assessing the permeability performance of PAC mixtures with different gradations.

## 1. Introduction

The development of asphalt pavements has increasingly emphasized serviceability and functionality. For conventional dense-graded asphalt pavements, water-related hazards such as splash and spray during rainy conditions can impair the visibility of following vehicles. At high driving speeds and under heavy rainfall, a water film can form between the tire and the pavement surface, significantly compromising driving safety and increasing the risk of traffic accidents. Statistics indicate that in China, approximately 25% of traffic accidents on the Beijing-Shijiazhuang highway occur during rainy or snowy weather conditions [1]. Research in Japan indicates that accident rates during rainfall are nine times higher than under dry conditions [2], while analysis from U.S. traffic data over a 30-year period found that precipitation contributed to over 25% of traffic accidents [3]. Additionally, the impervious nature of the dense-graded pavement contributes to rapid formation of the surface runoff during storms, potentially exacerbating urban flooding. The application of porous asphalt pavement has been recognized as an effective solution to these challenges [4].

Furthermore, rapid urbanization has led to the widespread expansion of impervious urban surfaces, which hinders the natural infiltration of rainfall and reduces groundwater recharge. In April 2012, the concept of the “Sponge City” was proposed in China to address urban waterlogging, enhance water resource utilization, protect aquatic environments, and mitigate the urban heat island effect [5]. Porous asphalt pavement, functioning as a natural facility for rainfall infiltration and purification, provides ecological benefits by facilitating the infiltration and storage of urban stormwater, thereby contributing to groundwater replenishment. Therefore, it plays a critical role in sponge city infrastructure [6,7].

Porous asphalt pavement is constructed using open-graded asphalt mixtures with the porosity typically exceeding 18% [8,9,10]. During rainfall events, these voids allow water to infiltrate through the pavement surface, thereby preventing the formation of water film. The skid resistance will be improved, water accumulation on pavement surface will be reduced, and the hydroplaning phenomenon will be prevented as well [6,11]. In addition, porous surfaces help to reduce road traffic noise [12]. These performance benefits stem from the interconnected void structure within the mixture. Therefore, maintaining a continuous and permeable void network is essential for ensuring effective drainage. However, due to variations in mixture composition, not all internal voids are interconnected. Water entering non-connected voids cannot be effectively discharged, thus failing to alleviate surface runoff [13,14]. It is therefore necessary to analyze the characteristics of effective voids under different air void levels in various mixture types.

The permeability performance of porous asphalt concrete (PAC) is commonly characterized by the permeability coefficient, with higher values indicating better water infiltration capability [15,16]. This performance is closely related to the void structure of the mixture—generally, greater void content leads to improved permeability. However, excessive void content may weaken the bonding between aggregates, further affecting durability and moisture resistance. Hence, a balance must be achieved between permeability and engineering performance to develop PAC mixtures with optimal structural and functional properties [17,18,19,20,21]. Most existing studies have focused on a single aspect of porous asphalt performance, often neglecting the integrated assessment of both functional (drainage) and structural (engineering) performance. Further investigations should be conducted to clarify and achieve this balance design [20,22,23,24,25].

In this context, this study focuses on PAC mixtures, specifically PAC-13 and PAC-10, as representative materials. Mix design and optimization were conducted for both types. A modified falling-head permeability test apparatus and procedures were developed to assess the permeability performance of PAC. The relationships between porosity and effective porosity were analyzed, and high-temperature stability and moisture susceptibility were evaluated. The variation in the permeability coefficient under different air void conditions was studied from a macro perspective, along with the influence of macrostructural parameters on the permeability performance of PAC. The findings provide theoretical support for the design and maintenance of porous asphalt pavements and are of significant value for promoting their practical application.

## 2. Materials and Methods

### 2.1. Raw Materials

Porous asphalt concrete (PAC) primarily consists of asphalt binder, a large proportion of coarse aggregates, a small amount of fine aggregates, and mineral filler. The properties of these raw materials play a critical role in determining the performance of PAC. Therefore, the selection of high-quality materials is essential.

**(1)**  **Asphalt Binder**

In this study, a SK70# base asphalt binder produced by SK Corporation (Seoul, Republic of Korea) was used, which was provided by Gansu Gongfa Longzhong High Tech Highway Maintenance Technology Co., Ltd., Lanzhou, China. The technical performance indicators of the asphalt binder are summarized in Table 1. It can be seen that all technical properties meet the requirements of the specification JTG E20-2011 [26].

**(2)**  **TPS High-Viscosity Modifier**

**Figure 1:**
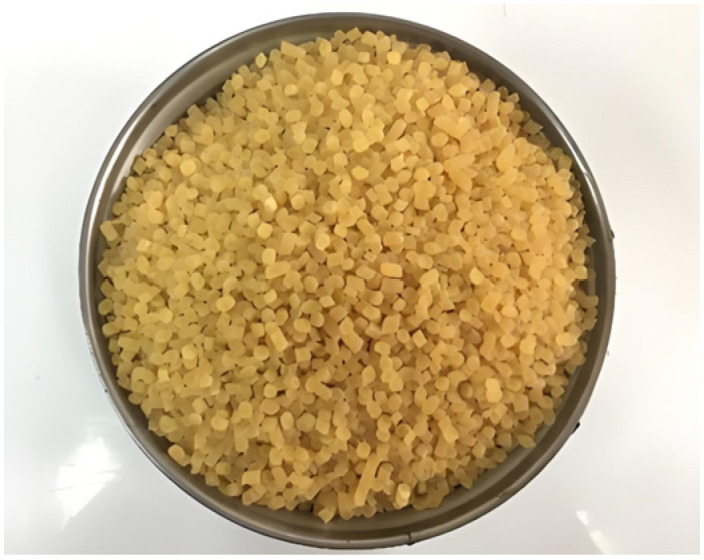
Appearance of TPS high-viscosity modifier.

To improve the bonding performance of the asphalt and mitigate raveling issues in porous asphalt mixtures, a high-viscosity modifier—TPS (TAFPACK-Super)—was selected. TPS is a specialized additive developed by DAIYU Co., Ltd., Shinjo, Japan, specifically for porous asphalt pavements. The primary component of TPS is thermoplastic rubber, with other ingredients including tackifying resins and plasticizers. It appears as yellow elastomeric granules measuring approximately 2–3 mm in diameter. Figure 1 shows the appearance of the TPS modifier, and its technical specifications are listed in Table 2.

**(3)**  **Coarse Aggregates**

High-quality basalt was selected as the coarse aggregate for this study, sourced from the Yongdeng Jianxin Quarry in Lanzhou of Gansu Province. The fundamental physical properties were tested according to JTG E42-2005 [27]. The results are presented in Table 3.

**(4)**  **Fine Aggregates**

Machine-manufactured sand from the same quarry was used as the fine aggregate. The basic physical properties were tested, and the results are shown in Table 4.

**(5)**  **Mineral Filler**

Limestone powder provided by Gansu Gongfa Longzhong Highway Maintenance Technology Co., Ltd., Lanzhou, China was used as the mineral filler in this study. The basic properties of the filler are summarized in Table 5.

### 2.2. PAC Mix Design

**Figure 2:**
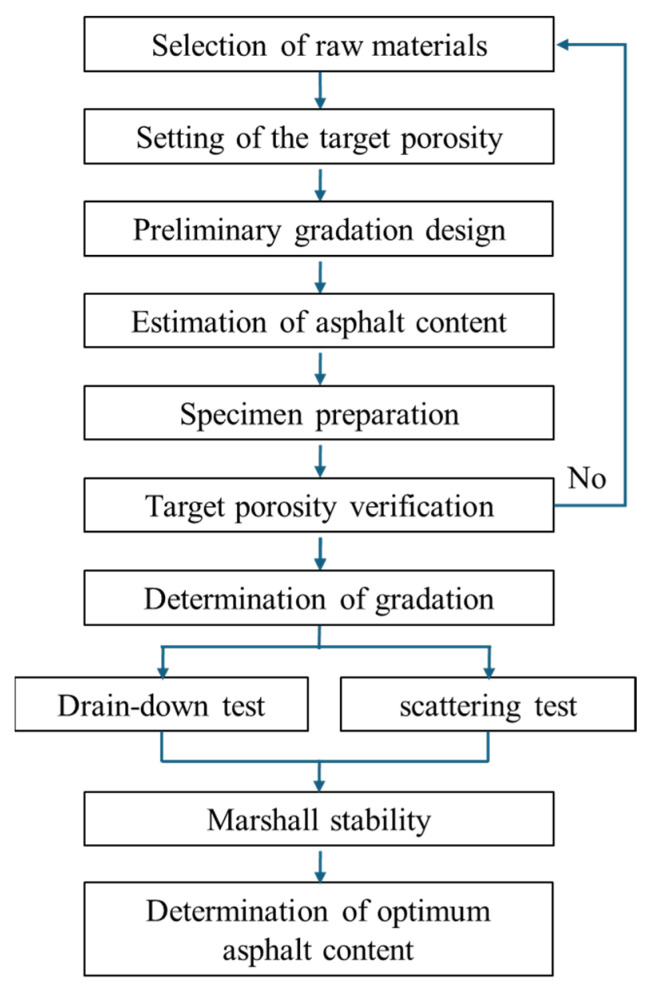
Procedures of the gradation curves determination for PAC-10 and PAC-13.

Two different nominal maximum aggregate sizes were used in this study, namely 13.2 mm (PAC-13) and 9.5 mm (PAC-10). The procedures of the gradation curves determination for PAC-10 and PAC-13 are shown in Figure 2. The key difference between the two gradations lies in the proportion of aggregates retained on each sieve, especially for the 9.5 mm and 4.75 mm sieves.

(1) Gradation design

In general, the permeability of porous asphalt concrete (PAC) increases with higher porosity. However, excessive air voids may compromise the durability of the mixture. Conversely, a void content that is too low may result in poor drainage performance and moisture retention, which could lead to water-induced damage. Therefore, the porosity of PAC is typically controlled within the range of 18% to 25% [28].

1. Preliminary gradation design

**Figure 3:**
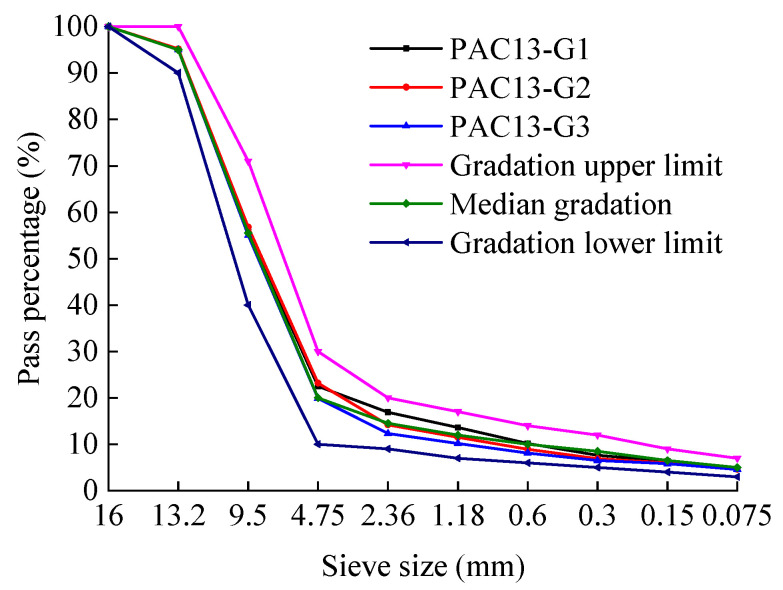
Preliminary gradation curves for PAC-13 mixtures.

**Figure 4:**
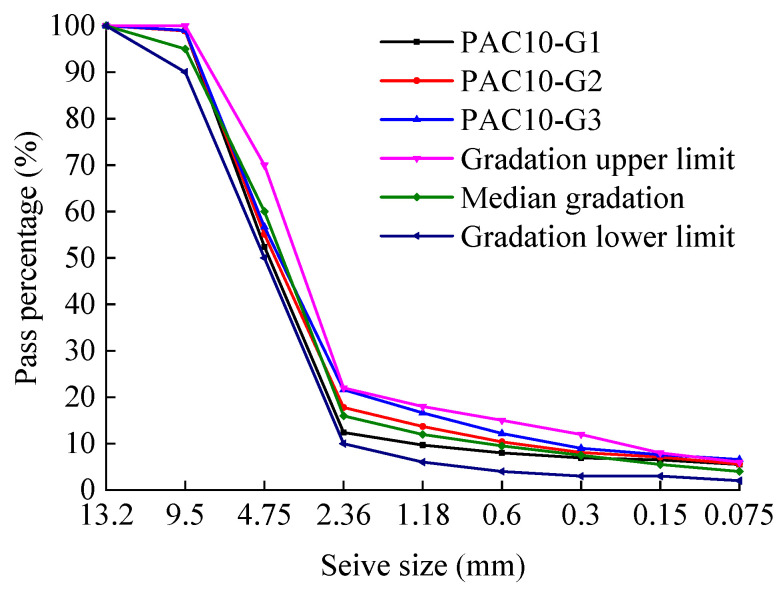
Preliminary gradation curves for PAC-10 mixtures.

Previous studies have shown that the porosity of PAC is closely related to the passing rate of the 2.36 mm sieve. Accordingly, three preliminary gradations were designed by adjusting the passing percentage of the 2.36 mm sieve within ±3% of the median value specified by the technical specifications. The corresponding gradation curves are shown in Figure 3 and Figure 4, respectively. G1 denotes the gradation where the passing percentage through the 2.36 mm sieve exceeds the median value by 3%, G2 represents the gradation where the passing percentage through the 2.36 mm sieve equals the median value, and G3 signifies the gradation where the passing percentage through the 2.36 mm sieve is below the median value by 3%.

2. Estimation of initial asphalt content

The asphalt content of PAC mixtures is typically estimated based on the surface area of the aggregate and its asphalt absorption capacity. Accordingly, the preliminary asphalt content for each gradation was calculated using Equation (1).

(1) $$P_{b}=h\times A$$

where *P_b_* is asphalt content, %; *h* is asphalt film thickness, μm; *A* is specific surface area of aggregates. The asphalt film thickness of 12 μm was assumed for PAC mixture, within the typical range of 10~14 μm [29].

The specific surface area *A* of the aggregates was calculated using Equation (2) [29].

(2) $$A=\left(0.41a+0.41b+0.82c+1.64d+2.87e+6.14f+12.29g+32.77h\right)/10^{3}$$

where *a*, *b*, *c*, *d*, *e*, *f*, *g*, and *h* represent the mass passing percentages through the 19 mm, 4.75 mm, 2.36 mm, 1.18 mm, 0.6 mm, 0.3 mm, 0.15 mm, and 0.075 mm sieves, respectively, %.

The estimated asphalt content for each PAC-13 and PAC-10 mixture are shown in Table 6.

3. Determination of target-porosity gradations

**Figure 5:**
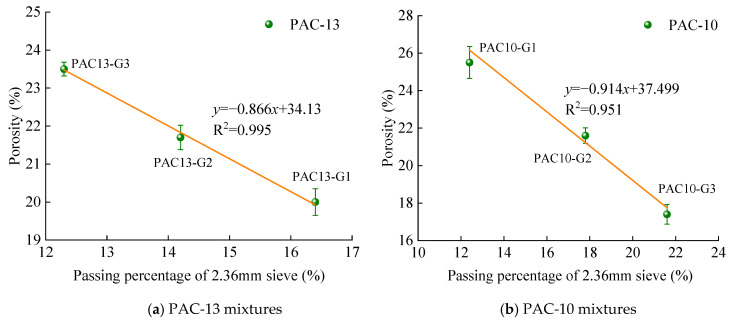
Relationship between 2.36 mm sieve passing percentage and porosity.

Using the gradation and asphalt content values in Table 3 and Table 4, Marshall specimens were prepared. Porosities were determined using volumetric measurement method. The volumetric results are shown in Table 7. The regression relationships between the 2.36 mm passing rate and porosity are plotted in Figure 5.

As illustrated in Figure 5, a strong linear correlation exists between the porosity and the 2.36 mm sieve passing percentage for both PAC-13 and PAC-10 mixtures, where the coefficient of determination R^2^ is larger than 0.95. This indicates that porosity can be predicted based on the 2.36 mm passing percentage, and conversely, desired void levels can be achieved by adjusting the 2.36 mm passing percentage. Based on this correlation, the passing rates corresponding to target porosities of 18%, 20%, and 25% were back-calculated, and the corresponding gradations were designed by adjusting the aggregate proportions. The results are summarized in Table 8.

**Figure 6:**
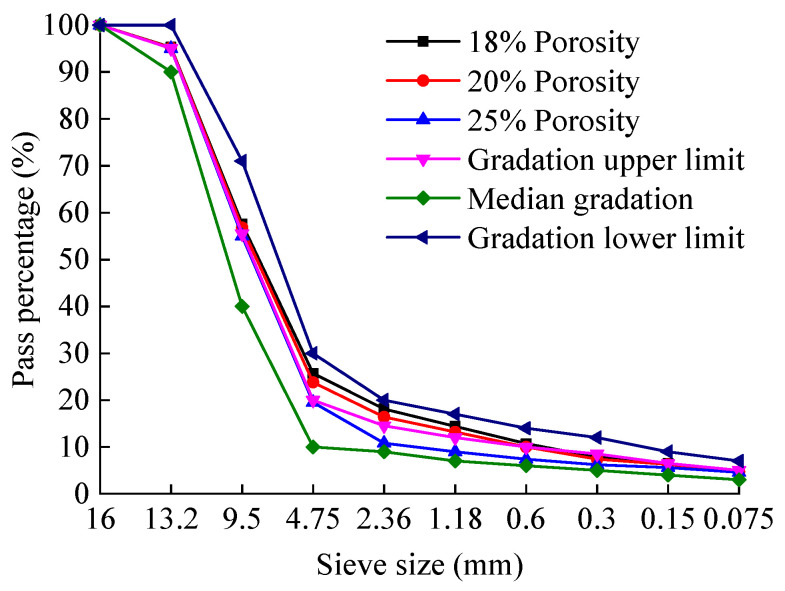
Gradation curves of PAC-13 mixtures with different target porosities.

**Figure 7:**
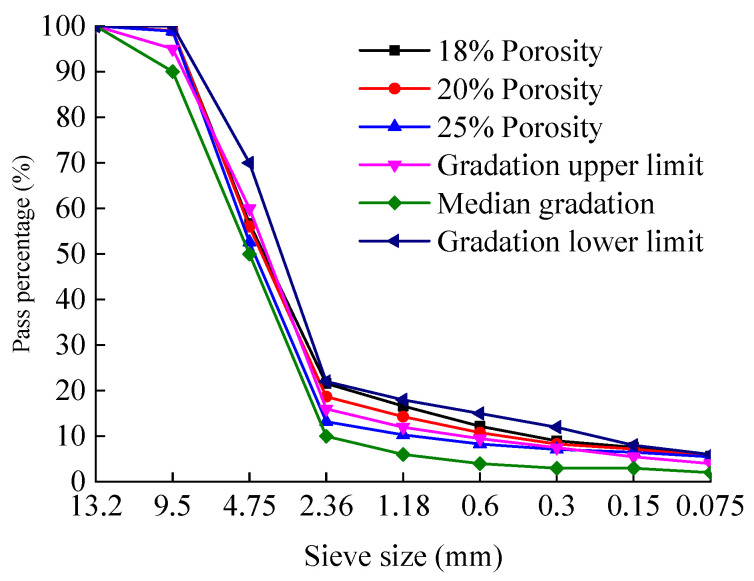
Gradation curves of PAC-10 mixtures with different target porosities.

Based on the 2.36 mm sieve passing percentage corresponding to each target porosity level for PAC-13 and PAC-10 in Table 8, and after appropriate adjustments, the final aggregate gradations were determined. The gradation curves are shown in Figure 6 and Figure 7, respectively.

(2) Determination of optimum asphalt content

The initial asphalt content was calculated using Equations (2) and (3), and the asphalt content varies by ±0.5% around this baseline value according to JTG E42-2005 [27]. Asphalt mixtures with different binder contents were prepared following the standard PAC mixing procedure. Subsequently, a binder drainage test, Cantabro test, and Marshall stability tests were conducted in accordance with relevant specifications. The optimum asphalt content range was determined based on the drainage test and Cantabro test results, while the final optimum content for each PAC mixture was selected with reference to the Marshall stability results.

The binder drainage test is used to identify the maximum allowable asphalt content that prevents binder segregation, whereas the Cantabro test determines the minimum binder content needed to avoid significant aggregate loss. In this study, the binder drainage test was performed using the beaker method as specified in the Specification JTG E20-2011 [26]. This method simulates asphalt drainage conditions during storage and transportation under elevated temperatures to evaluate the mixture’s resistance to drainage, where the drainage loss as a quantifiable indicator, defined as the mass ratio of drained asphalt binder to the total mixture, is calculated. The Cantabro test was conducted to evaluate the bonding strength between aggregates and binder, where the Cantabro loss, defined as the mass ratio of loss particles to the total mixture, was calculated according to JTG E20-2011.

**Figure 8:**
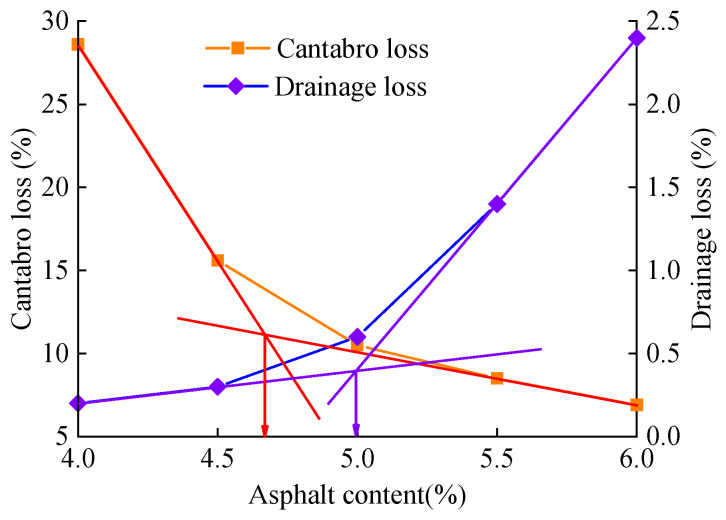
Relationship between asphalt content and drainage loss and Cantabro loss for PAC-13 mixture with 20% porosities.

The initial asphalt content for the PAC-13 mixture with a target porosity of 20% was calculated as 4.9% using Equation (1). Accordingly, five asphalt contents (4.0%, 4.5%, 5.0%, 5.5%, and 6.0%) were selected for testing. Figure 8 illustrates the relationship between asphalt content and both drainage loss and Cantabro loss. Moreover, Table 9 presents the corresponding porosities and Marshall stability values at different asphalt contents.

As shown in Figure 8, Cantabro loss decreases as the asphalt content increases, whereas drainage loss increases. The intersection points of the two curves indicate that the minimum and maximum allowable asphalt contents are 4.6% and 5.0%, respectively. Therefore, the optimum asphalt content range for PAC-13 with 20% target porosities was determined to be 4.6–5.0%. As presented in Table 9, the porosity decreases with increasing asphalt content. All mixtures tested exhibited Marshall stability values exceeding the minimum required standard of 3.5 kN. Considering the open-graded nature and durability requirements of PAC mixtures, a slightly higher asphalt content was selected to enhance the thickness of the asphalt film. Therefore, an optimum asphalt content of 4.9% was determined for the PAC-13 mixture with 20% target porosities.

The same procedure was employed to determine the optimum asphalt contents for all other PAC mixtures. The final optimum asphalt contents for PAC-10 and PAC-13 at different target porosity levels are summarized in Table 10.

### 2.3. Test Methods

(1) Preparation method of PAC specimen

According to the asphalt mixture specimen preparation method specified in the Specifications JTG E20-2011 [26], the porous asphalt concrete (PAC) was prepared as follows. It should be noted that the following used temperature and time are all from this specification.

Firstly, based on the pre-determined PAC gradation, a total of 1100 g of aggregate and mineral filler (by mass) was weighed according to each particle size category. The materials were then placed in an oven at 185 °C for 6 h for preheating.

Secondly, SK70# asphalt was placed in an oven at 135 °C for 1 h and then rapidly heated to 160 °C using an electric heater before mixing.

Thirdly, the preheated aggregates were added to a mixing pot preheated to 185 °C. A pre-measured amount of TPS modifier (at a ratio of TPS to base asphalt is 12 to 88) was added and dry mixed for 90 s.

Fourthly, after dry mixing, the preheated SK70# asphalt and mineral filler were added into mixing pot of mixing machine, followed by wet mixing for 90 s.

Fifthly, the prepared asphalt mixture was removed from the mixing pot and kept in an oven at 175 °C for 10 min. The mixture was then placed into a standard Marshall mold and compacted using a Marshall compactor with 50 blows on each side. The specimen was demolded after cooling for 24 h to ensure sufficient cooling to room temperature.

(2) Porosity measurement method of PAC specimen

The porosity of asphalt mixtures is generally controlled by the bulk volume density, apparent density, and maximum theoretical density. The Specifications JTG E20-2011 provide four methods for density measurement, namely the saturated surface-dry (SSD) method, immersion method, wax-sealing method, and volume method [26]. For PAC, the bulk volume density was measured using the volume method for the large porosity.

The bulk volume of a Marshall specimen is calculated by Equation (3).

(3) $$V=\frac{\pi \times d^{2}}{4}\times h$$

where *V* is bulk volume of the specimen, cm^3^; *d* is diameter of the Marshall specimen, cm; *h* is height of the specimen, cm.

The bulk density is calculated by Equation (4).

(4) $$\rho_{s}=\frac{m_{a}}{V}$$

where $\rho_{s}$ is bulk density, measured using the volume method, g/cm^3^; *m_a_* is mass of the dry specimen, g.

The bulk specific gravity at 25 °C is calculated by Equation (5).

(5) $$\gamma_{f}=\frac{\rho_{s}}{0.9971}$$

where $\gamma_{f}$ is bulk specific gravity, dimensionless.

The porosity *VV* is calculated by Equation (6).

(6) $$VV=\left(1-\frac{\gamma_{f}}{\gamma_{t}}\right)\times 100$$

where *VV* is porosity of the Marshall specimen, %.

**Figure 9:**
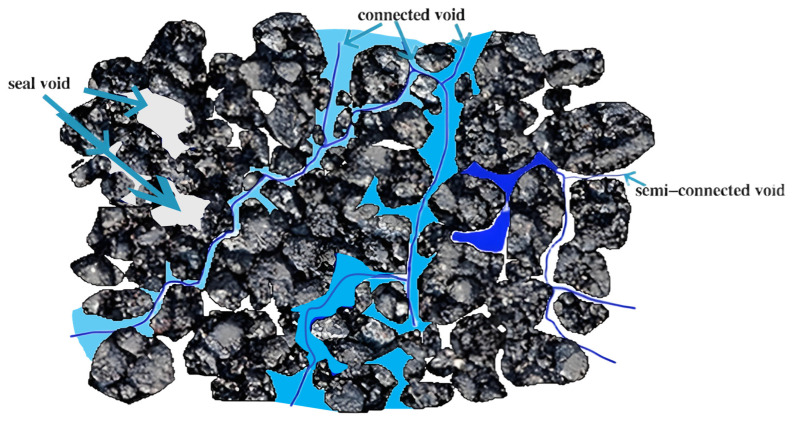
Schematic diagram of void distribution in porous asphalt mixture.

As shown in Figure 9, the voids calculated by Equation (6) include three types, namely connected voids, semi-connected voids, and seal voids. In practical water infiltration processes, only connected voids contribute to permeability, which was defined as the effective porosity.

The connected void is measured using the submerged method, as calculated by Equations (7) and (8).

(7) $$VV^{\prime }=\frac{V-V^{\prime }}{V}\times 100\%$$

(8) $$V^{\prime }=(A-C)/\rho_{w}$$

where $VV^{\prime }$ is the connected void volume, %; $V^{\prime }$ is the volume of the mixture excluding seal voids, mm^3^; V is the total volume of the specimen, mm^3^; A is the mass of the dry specimen, g; C is the mass of the specimen in water, g; $\rho_{w}$ is water density at room temperature, g/cm^3^.

(3) Permeability coefficient testing method for PAC

The permeability coefficient is derived based on Darcy’s law, which describes the inverse relationship between the flow velocity and the cross-sectional area of a pipeline under steady-state flow conditions when a fluid passes through it. The most commonly used measurement methods are the variable head test and the constant head test [30]. The constant head test is suitable for media with a large number of voids and a relatively high permeability coefficient. Such media usually require a relatively low starting pressure for the water flow. In the constant head test, a relatively small constant head difference is specified, and the permeability coefficient is measured by controlling the water flow velocity to ensure that the water flow state inside the medium is in a laminar flow state. The amount of seepage generated by the test specimen per unit time is measured. On the other hand, the variable head test is suitable for media with few voids and a low permeability coefficient. Such media require a relatively high starting pressure. The test is carried out by specifying an initial head height, and what is measured is the time required for a unit volume of water to pass through the test piece. The head is expressed as a function of time. The previous investigation found that the permeability coefficient of PAC mixtures can be measured with either a constant head method or a variable head method, while the variable head method mitigates measurement inaccuracies associated with low-flow-rate conditions [31].

**Figure 10:**
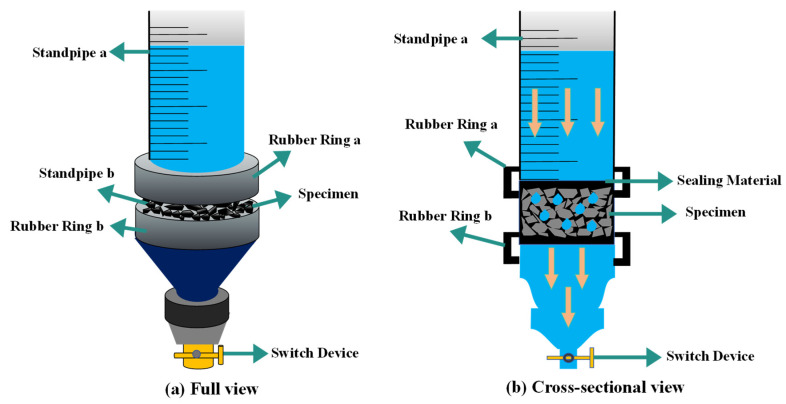
Schematic diagram of variable head permeameter: (**a**) Full view; (**b**) Cross-sectional view.

In this study, the variable head test method was used. Referring to the variable head permeability apparatus and method proposed in the literature [20], some modifications were made. A schematic diagram of the test setup is shown in Figure 10.

Prior to testing, the entire edge of the specimen was wrapped in plastic film. To seal any small gaps between the specimen and the inner wall of the standpipe, a silicone ring was added outside the plastic wrap, followed by another plastic film layer. Vaseline was applied to the outermost layer to prevent water leakage from the interface. Water was added until the level reached approximately 375 mm above the top surface of the specimen. After waiting for air to escape from the voids, the valve was opened. When the water level dropped to 300 mm above the specimen surface (*h*_1_), timing began. Timing stopped when the water level dropped to 75 mm above the specimen surface (*h*_2_). The permeability coefficient was then calculated using Darcy’s Law, as shown in Equation (9).

(9) $$K=\frac{aL}{At}\ln\frac{h_{1}}{h_{2}}$$

where *K* is permeability coefficient, mm/s; *a* is cross-sectional area of the standpipe, mm^2^; *L* is height of the specimen, mm; *A* is cross-sectional area of the specimen, mm^2^; *t* is permeability time, s.

## 3. Results and Discussions

### 3.1. Analysis of Porosity and Effective Porosity in PAC

**Figure 11:**
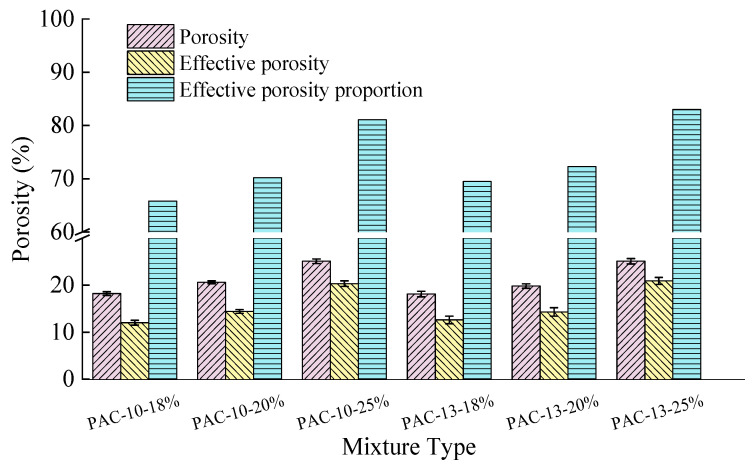
Variations in effective porosity and effective porosity proportion with different mixtures.

Based on the porosity and effective porosity testing methods described in Section 2.3, the porosity of PAC-10 and PAC-13 at various porosity levels was measured. The variations in porosity and effective porosity with different mixtures are shown in Figure 11. Moreover, the effective porosity proportion was also calculated, which refers to the ratio of effective porosity to porosity. Three replicate specimens for different mixture types were prepared and evaluated.

As shown in Figure 11, both the effective porosity and the effective porosity proportion in PAC-10 and PAC-13 increase with increasing porosity. However, for the same porosity, PAC-13 consistently exhibits a higher effective porosity proportion compared to PAC-10, which is about 2% to 3% higher. This difference is primarily due to the variations in aggregate gradation, which results in different internal void structures in the asphalt mixtures, influencing the voids filled with asphalt (VFA) and the differences in filling status. Specifically, the PAC-13 mixture contains a higher proportion of coarse aggregates, resulting in enlarged void spaces within the mixture and consequently increasing the effective porosity proportion. The PAC-10 mixture exhibits smaller void spaces alongside higher asphalt content from Table 10, resulting in increased VFA and a consequent reduction in effective porosity [32].

### 3.2. Engineering Performance Evaluation of PAC

(1) High-temperature stability test results

**Figure 12:**
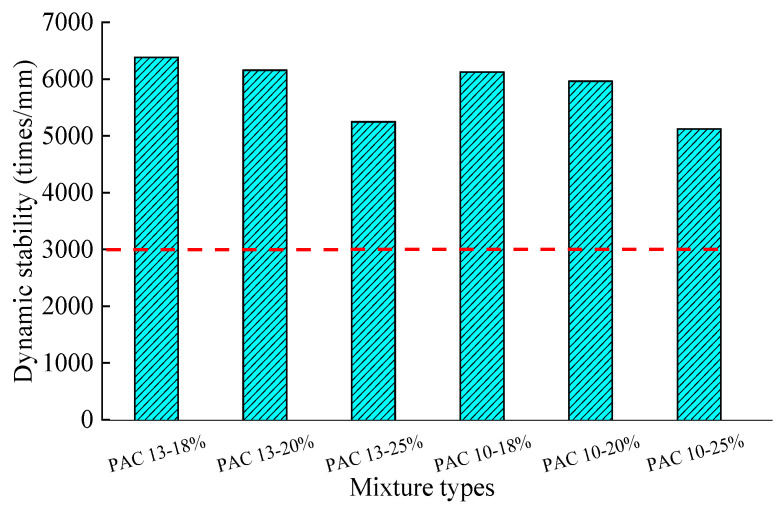
Variations in dynamic stability with different mixtures and porosities.

A rutting test was conducted to evaluate the high-temperature performance of PAC. The variations in dynamic stability with different mixtures and porosities are shown in Figure 12. Three replicate specimens were prepared and evaluated. Results with coefficients of variation below 20% were averaged.

As observed in Figure 12, both the porosity and nominal maximum aggregate size (NMAS) significantly affect the high-temperature stability of PAC. For mixtures with the same porosity, specimens with a 13.2 mm NMAS exhibit slightly better rutting resistance than those with a 9.5 mm NMAS. Specifically, the PAC-13 mixture exhibits 2.5–3.3% higher dynamic stability than PAC-10. Additionally, for mixtures with the same NMAS, the dynamic stability decreases with increasing porosity. Compared to Figure 11, it can be found that large porosity and high-temperature stability are contradictory, which needs to be balanced in the future. For example, a stable skeletal structure predominantly composed of highly angular coarse aggregates can be constructed, a gap-graded design strategy is employed. This approach significantly reduces intermediate-sized particles while strictly controlling fine aggregate and filler content to mitigate skeletal void clogging. Void interconnectivity is thereby preserved through the utilization of high-performance asphalt binders, enabling optimization of binder content. However, all PAC specimens meet the dynamic stability requirement of considerably larger than 3000 cycles/mm, indicating that the PAC mixtures used in this study exhibit excellent rutting resistance [15,33].

(2) Moisture susceptibility test results

**Figure 13:**
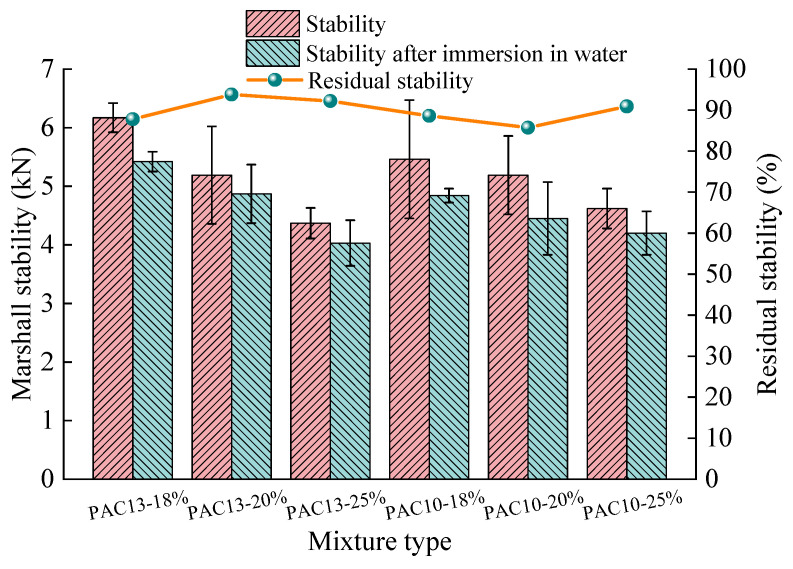
Immersion Marshall test results for PAC with different mixtures and porosities.

The moisture susceptibility of PAC with different mixtures and porosities was evaluated using the immersion Marshall test, and the results are presented in Figure 13. Three replicate specimens were prepared and evaluated.

From the immersion Marshall test results shown in Figure 13, both PAC specimens before and after immersion show a decrease in Marshall stability as porosity increases. However, all values exceed the minimum standard requirement of 3.5 kN. Additionally, the retained stability is greater than 85% for all specimens, indicating that the PAC mixtures studied in this research have excellent resistance to moisture damage.

### 3.3. Permeability Performance Analysis of PAC

(1) Effect of nominal maximum aggregate size on permeability of PAC

**Figure 14:**
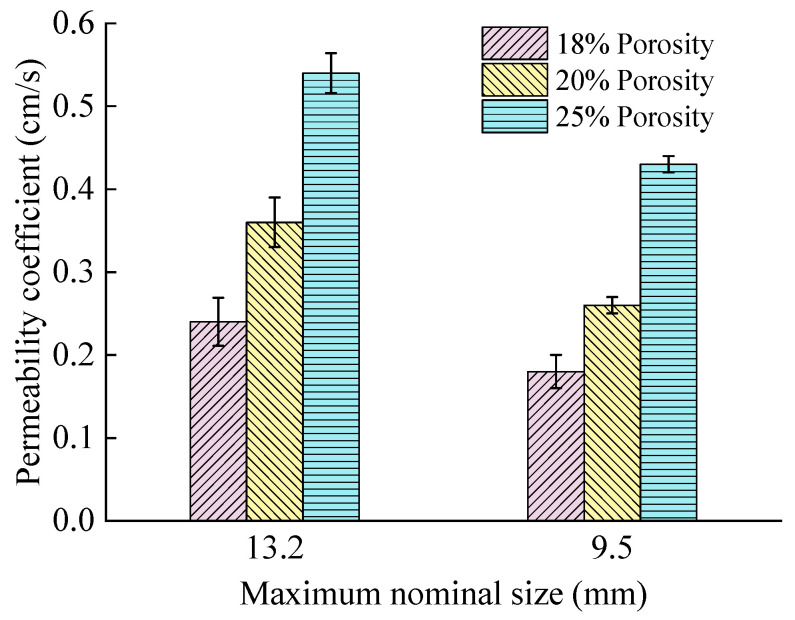
Effect of NMAS on the initial permeability coefficient of PAC.

The permeability coefficients for PAC with different NMAS values were measured, and the results are shown in Figure 14. Three replicate specimens for different mixture types were prepared and evaluated.

As illustrated in Figure 14, PAC mixtures with different NMAS values exhibit significant differences in their initial permeability coefficients. The measured values were considered reliable, as evidenced by a coefficient of variation (CV) below 3%. For porosities of 18%, 20%, and 25%, PAC with a 13.2 mm NMAS demonstrates initial permeability coefficients that are 33.3%, 38.5%, and 25.5% higher, respectively, than those for PAC with a 9.5 mm NMAS. This indicates that mixtures with larger NMAS exhibit higher water permeability. Based on the porosity test results, PAC-13 has a higher effective porosity proportion than PAC-10 at the same porosity level. Furthermore, PAC-13 contains a larger proportion of 10–15 mm aggregates, which form a more pronounced interlocking structure compared to smaller aggregates, leading to enhanced permeability with larger NMAS [34].

(2) Effect of porosity on permeability of PAC

**Figure 15:**
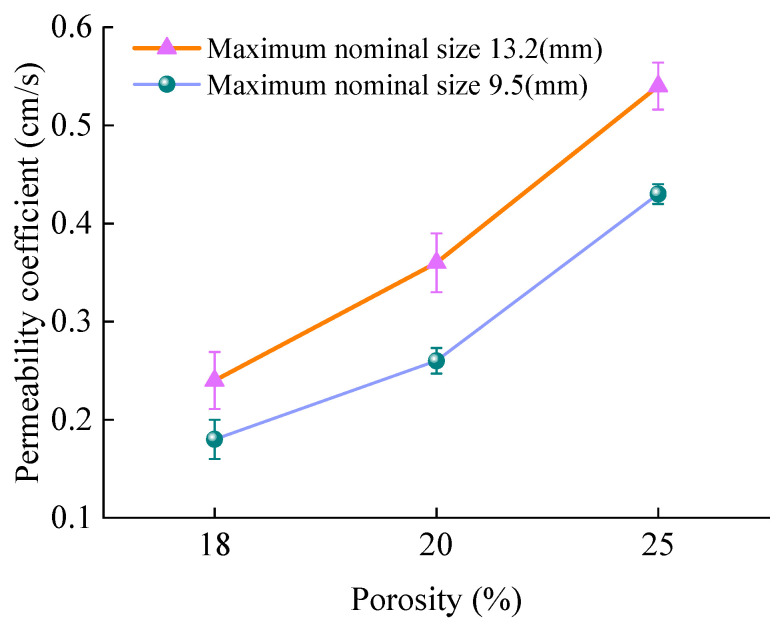
Effect of porosity on the initial permeability coefficient of PAC.

The effect of porosity on the permeability coefficient of PAC was analyzed, and the results are shown in Figure 15.

**Figure 16:**
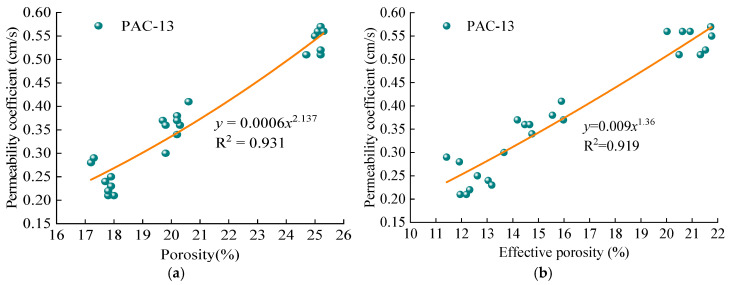

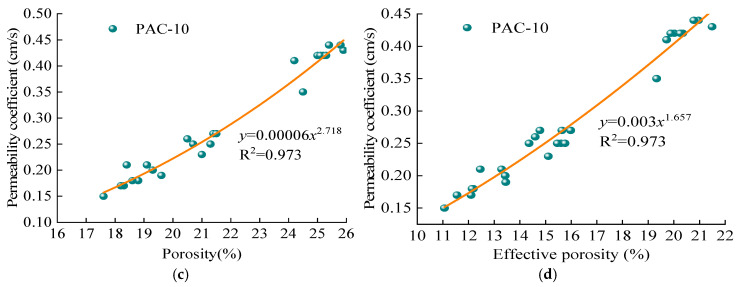
Relationship between permeability coefficient and (**a**) porosity for PAC-13; (**b**) effective porosity for PAC-13; (**c**) porosity for PAC-10; (**d**) effective porosity for PAC-10.

As depicted in Figure 15, the permeability coefficient of PAC increases as porosity rises. For PAC with a 13.2 mm NMAS, the initial permeability coefficients at 20% and 25% porosity are 50% and 125% higher, respectively, than that at 18%. For PAC with a 9.5 mm NMAS, the corresponding increases are 44.4% and 139%, respectively. This demonstrates a positive correlation between the permeability coefficient and both porosity and effective porosity. Consequently, a power function model (Equation (10)) was used to regress the relationship between the permeability coefficient and (effective) porosity for both PAC-10 and PAC-13. The regression results are presented in Figure 16.

(10) $$k=aV^{b}$$

where *a* and *b* are regression coefficients; *V* is (effective) porosity of the PAC specimen, %.

As shown in Figure 16, the permeability coefficient increases with increasing porosity for both PAC-10 and PAC-13. The coefficient of determination (R^2^) for PAC-10 and PAC-13 is 0.919 and 0.937, respectively, indicating a strong power function relationship. Furthermore, the correlation between effective porosity and the permeability coefficient is stronger than that between total porosity and permeability. This is because only effective porosities contribute to water storage and drainage in PAC, consistent with the findings of most researchers [35,36,37].

## 4. Conclusions

This study used PAC-13 and PAC-10 porous asphalt mixtures to prepare specimens with varying porosities. The porosity characteristics and pavement performance of these mixtures were analyzed, and the effects of nominal maximum aggregate size and porosity on the permeability coefficient were examined. The main conclusions are as follows.

(1) Both effective porosity and the effective porosity proportion increase with rising porosity in PAC-10 and PAC-13. PAC-13 consistently exhibits a higher effective porosity proportion than PAC-10 at the same porosity level. Therefore, PAC-13 is recommended for improved drainage performance.
(2) Based on the high-temperature stability and moisture susceptibility test results, the PAC mixtures used in this study meet the requirements for asphalt pavement performance, while the large porosity is contradictory with high-temperature stability and moisture resistance.
(3) Significant differences in permeability coefficients were observed for PAC mixtures with different nominal maximum aggregate sizes. The permeability coefficient increases with increasing NMAS. Moreover, the permeability coefficient increases with higher porosity, and a strong linear correlation exists between the permeability coefficient and both porosity and effective porosity. Thus, these void parameters can be used as indirect indicators of the water permeability performance of PAC mixtures.

The contradiction between high porosity and high temperature and moisture resistance can be observed in this study. Therefore, the design of highly permeable asphalt pavement needs to be further investigated by balancing permeable performance and engineering performance. Some attempts can be made to optimize the skeleton structures to enable the favorable interlocking of coarse aggregate, promote the interconnected void networks, enhance binder cohesion strength, further reduce asphalt content, etc.

## Figures and Tables

**Table 1:** Technical properties of SK70# asphalt binder.

Technical Indicators		Unit	Measured Value	Required Value	Test Method
Penetration (25 °C, 100 g, 5 s)		0.1 mm	68.7	60~80	T0604
Softening point		°C	46.8	≥46	T0606
Ductility (10 °C)		cm	41.7	≥20	T0605
Ductility (15 °C)		cm	>150	≥100	
Density		g/cm^3^	1.032	Measured value	T0603
Dynamic viscosity (60 °C)		Pa·s	191	≥180	T0620
After RTFOF (163 °C, 85 min)	Mass change	%	0.12	≤±0.8	T0610
	Residual penetration ratio (25 °C)	%	68	≥65	T0604
	Residual ductility (10 °C)	cm	6.2	≥6	T0605

**Table 2:** Technical Specifications of TPS Modifier.

Indicators	Shape	Color	Specific Gravity	Weight per Unit Volume
Result	Granular (3–5 mm)	Light yellow	0.98	0.6 t/m^3^

**Table 3:** Technical properties of coarse aggregates.

Technical Indicators		Unit	Test Results	Required Value	Test Method
Bulk relative density	10~15 mm	g/cm^3^	2.774	≥2.6	T0304
	5~10 mm		2.758	≥2.6	
	3~5 mm		2.728	≥2.6	
Apparent relative density	10~15 mm	g/cm^3^	2.804	≥2.6	
	5~10 mm		2.804	≥2.6	
	3~5 mm		2.785	≥2.6	
Water absorption rate	10~15 mm	%	0.384	≤2.0	T0307
	5~10 mm		0.592	≤2.0	
	3~5 mm		0.761	≤2.0	
Crushing value		%	13.8	≤26	T0316
Los Angeles abrasion loss		%	18.3	≤28	T0323
Soundness		%	1.4	≤8	T0314
Adhesion with asphalt binder		Grade	5	5	T0654
Flaky and elongated particle contents	Particle size > 9.5 mm	%	5.3	≤10	T0312
	Particle size < 9.5 mm		4.7	≤12	
<0.075 mm content		%	0.4	≤1	T0310

**Table 4:** Technical properties of fine aggregates.

Technical Index	Unit	Test Result	Specification	Test Method
Apparent relative density	g/cm^3^	2.785	≥2.5	T0330
Sand equivalent	%	81.6	≥60	T0334
Angularity	s	56.3	≥30	T0345
Soundness	%	16.7	≥10	T0340
<0.075 mm Sieve	%	0.84	≤1	T0333

**Table 5:** Technical properties of mineral filler.

Technical Indicators	Unit	Test Results	Required Value	Test Method
Apparent relative density	g/cm^3^	2.735	≥2.5	T0352
Appearance	-	No agglomerates or caking	No agglomerates or caking	-
Hydrophilic coefficient	-	0.72	<1	T0353
Plasticity index	%	2.2	<4	T0354
Moisture content	%	0.18	≤1	T0103
Heat stability	-	No change	Measured record	T0355
<0.6 mm	%	100	100	T0351
<0.15 mm	%	98.88	90~100	
<0.075 mm	%	90.55	75~100	

**Table 6:** Estimated asphalt contents for preliminary gradations.

Mixture types	PAC13-G1	PAC13-G2	PAC13-G3	PAC10-G1	PAC10-G2	PAC10-G3
Asphalt contents (%)	5.0	4.7	4.5	5.2	5.5	5.8

**Table 7:** Volumetric properties of PAC-13 and PAC-10 specimens.

Specimen ID	Maximum Theoretical Relative Density (g/cm^3^)	Bulk Relative Density (g/cm^3^)	Porosity (%)
PAC13-G1	2.591	2.072	20.0
PAC13-G2	2.598	2.034	21.7
PAC13-G3	2.607	1.994	23.5
PAC10-G1	2.571	1.915	25.5
PAC10-G2	2.552	2.002	21.6
PAC10-G3	2.540	2.098	17.4

**Table 8:** Passing percentages of 2.36 mm sieve corresponding to target porosity levels.

Target Porosity (%)	2.36 mm Sieve Passing Percentage (%)	
	PAC-13	PAC-10
18	18.6	21.3
20	16.3	19.1
25	10.5	13.7

**Table 9:** Porosities and Marshall stability for PAC-13 mixtures (target porosities equal 20%) at different asphalt contents.

Asphalt Content (%)	Porosities (%)	Marshall Stability (kN)
4.0	21.9	5.19
4.5	21.2	6.06
5.0	20.5	6.63
5.5	19.6	5.64
6.0	18.8	8.33

**Table 10:** Optimum asphalt contents for PAC mixtures at different target porosities.

Target Porosities (%)	Optimum Asphalt Content (%)	
	PAC-13	PAC-10
18	5.1	5.8
20	4.9	5.5
25	4.5	5.3

## Data Availability

The original contributions presented in this study are included in the article; further inquiries can be directed to the corresponding author.
